# Decreased trend in hospital mortality from pancreatic cancer despite increase in number of hospital admissions

**DOI:** 10.1371/journal.pone.0199909

**Published:** 2018-07-18

**Authors:** Sanjay Bhandari, Mubeen Khan Mohammed Abdul, Will Hollabaugh, Kanav Sharma, Douglas B. Evans, Nalini Guda

**Affiliations:** 1 Division of General Internal Medicine, Department of Medicine, Medical College of Wisconsin, Milwaukee, WI, United States of America; 2 Department of Medicine, Aurora Saint Luke’s Medical Center, Milwaukee, WI, United States of America; 3 Medical College of Wisconsin, Milwaukee, WI, United States of America; 4 Division of Surgical Oncology, Department of Surgery, Medical College of Wisconsin Milwaukee, WI, United States of America; 5 Division of Gastroenterology, Aurora Saint Luke’s Medical Center, Milwaukee, WI, United States of America; Augusta University, UNITED STATES

## Abstract

**Background and aim:**

Pancreatic cancer is one of the common cancers in US and is associated with high mortality and morbidity. The objectives of our study were to look at the recent trends in the number of hospitalizations with pancreatic cancer.

**Methods:**

We identified patients with a discharge diagnosis of pancreatic cancer in the National Inpatient Sample from 2007 to 2011 using International Classification of Diseases—Clinical Modification, 9th revision (ICD-9-CM) codes. We looked at the yearly trend in the hospitalizations with pancreatic cancer and the outcomes which included length of stay (LOS), hospital charges and in-hospital mortality. We also performed multivariate analysis to look for the predictors of mortality.

**Results:**

There were 450, 414 patients with discharge diagnosis of pancreatic cancer. There was 18% increase in hospitalizations with pancreatic cancer in 2011 compared to 2007. Most of the patients were Caucasian (63%) with the mean age of 68 ± 0.14 years, had Medicare (57%) as primary insurance, were from Southern region (35%) and had higher Charlson Comorbidity Index (CCI) (87% with CCI > = 5). 6% underwent Whipple’s procedure in the index hospitalization. After the adjustment for inflation, the mean hospital charges increased from $ 47,331 in 20007 to $ 53, 854 in 2011 (p = 0.01). LOS decreased from 7.31 ± 0.11 days in 2007 to 6.70 ± 0.09 days in 2011 (<0.001). Despite the increase in the number of hospitalizations of patients with pancreatic cancer, mortality decreased from 9.8% in 2007 to 8.1% in 2011 (p<0.001). On multivariate analysis, the independent factors associated with higher mortality were older age, male sex African-American race, insurance status other than Medicare, higher CCI and enrollment in palliative care. There was regional variation in mortality. Whipple’s procedure conferred lower mortality.

**Conclusions:**

Our study showed downward trends in LOS and in-hospital mortality despite increasing hospitalizations with pancreatic cancer.

## Introduction

Pancreatic cancer is the 12^th^ most common cause of cancer in United States, and low survival rates make it the 4^th^ most common cause of cancer-related deaths in both men and women [1, 2]. Years of life lost (YLL), which acts as an indirect assessment of the number of estimated years of life lost due to premature mortality, establishes pancreatic cancer as the 20th leading cause of years of life lost in the United States due to all causes of mortality, in both sexes and all races combined [3]. Further, with an aging population and changing demographics, incidence of pancreatic cancer is expected to increase over time and is projected to be the 2^nd^ most common cause of cancer-related deaths by 2030 [4].

Pancreatic cancer is associated with high morbidity and mortality. Approximately 53,000 Americans were anticipated to be diagnosed with pancreatic cancer and 42,000 were anticipated to die from this disease in 2016 [2]. The 5-year survival rate from pancreatic cancer between 2005 and 2011 was approximately 8% and the survival has improved only slightly in last 35 years [2]. Approximately 20% of the patients who undergo surgery would survive from the cancer. Unfortunately, only 15–20% of pancreatic cancer patients are candidates for surgery [5, 6]. The most common surgery performed is the curative-intent pancreaticoduodenectomy, or Whipple procedure, which is associated with high morbidity, prolonged in-hospital stay and has a mortality rate of 1–5% [7]. Patients who undergo surgery may receive adjuvant chemotherapy after surgery, and patients with unresectable tumors may be candidates for chemotherapy or chemo radiation therapy; all of these contribute significantly to morbidity [8, 9].

Pancreatic cancer patients have frequent hospitalizations for establishment of diagnosis, recovery from surgery and for complications related to cancer, surgery or chemotherapy [10, 11]. Hospital admissions are associated with decreased quality of life and increased cost of care. In the United States, little information is known to date about the profile of pancreatic cancer patients of all insurance statuses who are admitted to hospitals, their length of stay or the economic impact of their hospitalizations. Our goal was to identify overall trends regarding pancreatic cancer within the United States. Our objective also implies that the information obtained will lead to future discussions and investigations regarding the need for advances in pancreatic cancer treatment.

## Methods and materials

### Data source and study population

National Inpatient Sample (NIS), which is the largest all-payer inpatient care database publicly available in the United States, was used for this study. The NIS is an administrative dataset created by the Agency for Healthcare Research and Quality from a 20% stratified sample of U.S. community hospitals [12]. Each hospitalization is treated as an individual entry in the database and is coded with 15 to 25 diagnosis (15 prior to the 2009 NIS) and 15 procedural diagnoses. Both hospital and discharge weights are provided to facilitate the production of national estimates.

Hospital discharge data from the NIS from year 2007 to 2011 was used for the study. Using the International Classification of Diseases—Clinical Modification, 9th revision (ICD-9-CM) codes, all adult patients aged 18 years or more with the primary diagnosis of pancreatic cancer (ICD-9-CM code 157.*) were queried. All the clinical characteristics of the patients, hospital outcomes and yearly trend of hospitalizations from pancreatic cancer were assessed. We performed the multivariate logistic regression to determine the independent risk factors of mortality.

The demographic information on pancreatic cancer related hospitalizations included age, sex, race, primary insurance and hospital locations. Deyo’s modification of Charlson’s Comorbidity Index (CCI) was used for identifying the burden of co-morbid disease [13]. Further, we also included information on Whipple procedure performed during the index hospitalization.

### Statistical analysis

The discharge weights were used to generate national estimates. The categorical variables were presented as percentages, and numeric variables such as age, hospital charges and length of stay as means. Categorical variables were compared using Chi-square test through SURVEYFREQ procedure. Similarly, continuous variables were compared using *t-test* through SURVEYREG procedure. Multivariable logistic regression model was used to study the independent predictors for in-hospital mortality. Rates of hospitalizations with pancreatic cancer were adjusted for population growth as obtained from the US Census Bureau (https://www.census.gov/en.html). Adjusted rate of hospitalization for any given year was calculated by dividing the total hospitalizations with pancreatic cancer from the NIS data by the population estimate obtained from the US Census Bureau for that year. Similarly, the hospital charges were adjusted for inflation using the data from the U.S. Bureau of Labor Statistics (https://www.bls.gov/data/inflation_calculator.htm). Statistical significance was defined as a p-value < 0.05. Statistical analysis was performed using SAS 9.4 (Cary, NC: SAS Institute Inc).

## Results

### Demographic characteristics

Within the National Inpatient Sample from 2007 to 2011, there were 450,414 patients (weighted for national estimate) with a discharge diagnosis of pancreatic cancer. The clinical characteristics of the patients included in the study are shown in Table 1. The mean age of the population was 67.96 years with the median age of 67.83 years. The majority of patients were white (63%), female (51%), had Medicare (57%) as their primary insurance, live in the Southern region of the United States (35%) and had higher Charlson Comorbidity Index (CCI) (87% with CCI > = 5). During the index hospitalization, 6% of patients included in this study underwent the Whipple procedure and 8% enrolled in palliative care.

**Table 1:** Characteristics of patients admitted for pancreatic cancer.

Covariates	Pancreatic Cancer (N = 450,414) %
Age, yrs Mean (67.96 ± 0.14) Median (67.83 ± 0.16)	
Age Category, yrs	
**18–40**	2
**41–60**	26
**61–80**	55
**> = 81**	17
**Sex**	
**Female**	50.5
**Male**	49.5
**Race**	
**White**	63
**Black**	11
**Hispanic**	6
**Others/Missing**	20
**Primary Insurance Payer**	
**Medicare**	57
**Medicaid**	7
**Private Insurance**	31
**Self-Pay**	2
**No Charge**	<1
**Others/Missing**	3
**Hospital Region**	
**Northeast**	23
**Midwest**	23
**South**	35
**West**	19
**Charlson Comorbidity Index**	
**< = 2**	2
**3**	4
**4**	7
**> = 5**	87
**Whipple Procedure (during index hospitalization)**	6
**Palliative Care Encounter**	8

### Trends in pancreatic cancer related hospitalizations and associated hospital outcomes

Our analysis exhibited an 18% increase in hospitalizations due to pancreatic cancer from 82,309 in 2007 to 97,016 in 2011 (Table 2). A separate analysis looking into individual trend pertaining to different age groups showed that there was 11% decrease in hospitalizations in the age group of 18–40 years (from 1,588 in 2007 to 1,418 in 2011), 13% increase in the age group of 40–60 years (from 21,663 in 2007 to 24,405 in 2011), 20% increase in the age group of 60–80 years (from 45,043 in 2007 to 54,221 in 2011) and 21% increase in the age group of > = 81 years (from 14,016 in 2007 to 16,971 in 2011).

**Table 2:** Trends in the outcomes of hospital admissions with pancreatic cancer.

Outcomes	2007 (N = 82,309)	2008 (N = 89,452)	2009 (N = 86,806)	2010 (N = 94,832)	2011 (N = 97,016)	P-values for trend
**Died (%)**	9.8	9.8	9.4	8.3	8.1	<0.001
**Mean Hospital**	47,331	49,430	50,630	53,521	53,854	0.012
**Charges (Dollars)**	45,979 ^a^	48,067 ^a^	49,113 ^a^	52,339 ^a^	52,549 ^a^	0.007 ^a^
**Mean Length of**	7.3	7.1	6.9	6.8	6.7	<0.001
**Stay (Days)**	7.2 ^a^	7.0 ^a^	6.8 ^a^	6.8 ^a^	6.6 ^a^	<0.001^a^

^a^ With exclusion of those who died in the hospital.

**Fig 1:**
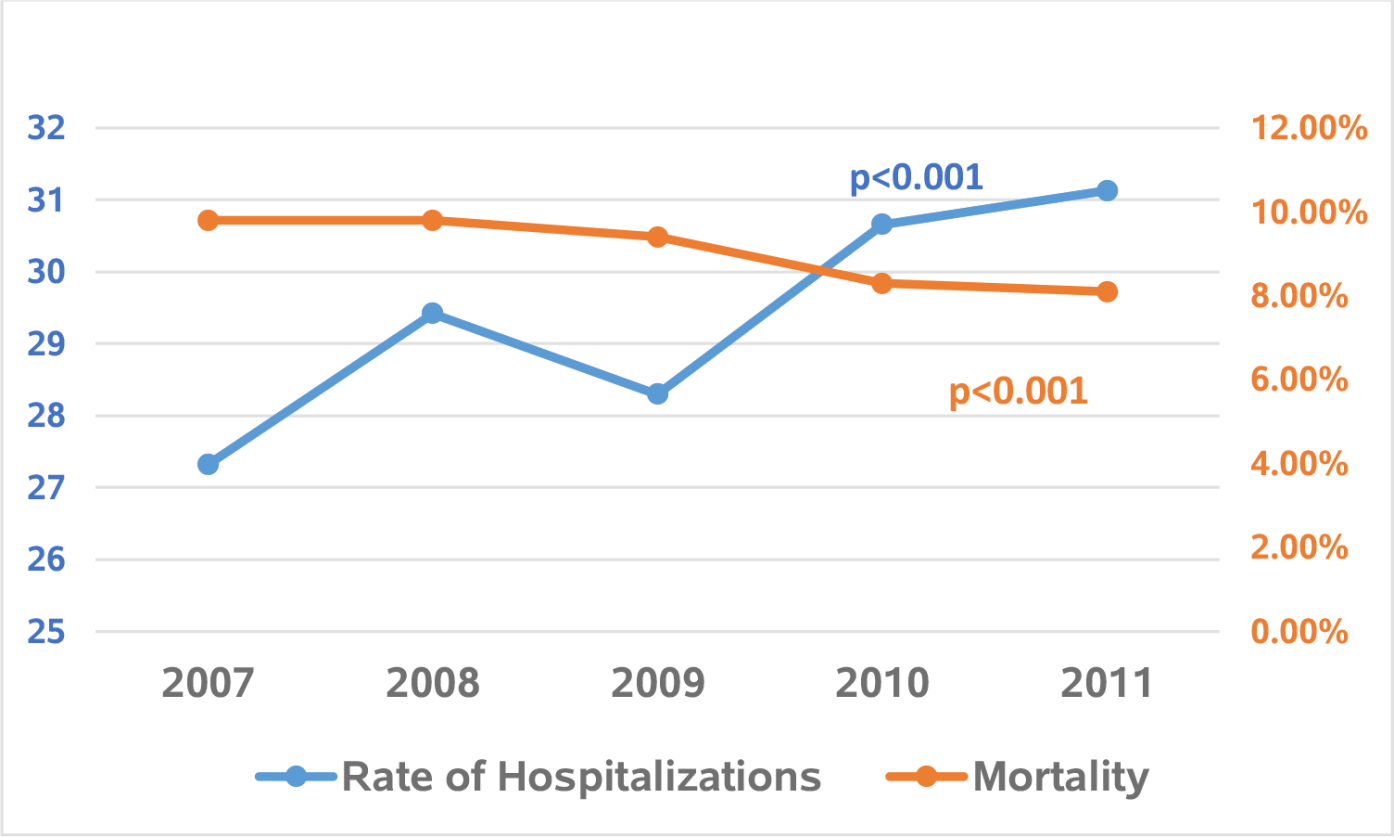
Trends in adjusted rates of hospitalizations (per 100,000 US population) and in-hospital mortality in pancreatic cancer.

After adjusting for yearly population growth as obtained from the US Census Bureau, the hospitalization rates of pancreatic cancer per 100,000 US population were 27.32 in 2007, 29.42 in 2008, 28.30 in 2009, 30.66 in 2010 and 31.13 in 2011 (p-value <0.001), reflecting an overall increase of 14% from 2007 to 2011 (Fig 1).

Despite the increase in the number of hospitalizations due to pancreatic cancer, in-hospital mortality rate decreased from 9.8% in 2007 to 8.1% in 2011 (p<0.001) (Table 2 and Fig 1). On multivariate analysis, the independent predictors of higher mortality were older age (> = 81 vs 18–40 years, OR: 1.9, 95% CI 1.5–2.5), male sex (OR 1.2, 95% CI 1.1–1.2), Black race (vs White: OR 1.3, 95% CI 1.2–1.4), insurance status other than Medicare, and higher CCI scores (score of > = 5 with the highest odds: OR 2.6, 95% CI 2.0–3.5) (Table 3). Patients who were enrolled in palliative care had higher odds of in-hospital death (OR 6.5, 95% CI 6.0–7.1). In terms of geographical location, regions other than Northeast demonstrated lower mortality. Patients admitted for the Whipple procedure (OR 0.6, 95% CI 95% 0.5–0.7, p<0.0001) expectedly demonstrated lower mortality (Table 3).

**Table 3:** Predictors of in-hospital mortality in pancreatic cancer on multivariate analysis.

Covariates	Odds-Ratios	95% Confidence Limits		P-Values
**Age-Category**				
** • 18–40**	Ref	Ref	Ref	Ref
** • 41–60**	1.484	1.151	1.914	0.002
** • 61–80**	1.729	1.342	2.227	<0.001
** • > = 81**	1.936	1.489	2.517	<0.001
**Sex**				
** • Female**	Ref	Ref	Ref	Ref
** • Male**	1.187	1.129	1.248	<0.001
**Race**				
** • White**	Ref	Ref	Ref	Ref
** • Black**	1.275	1.167	1.393	<0.001
** • Hispanic**	1.104	0.981	1.242	0.101
** • Others/Missing**	1.322	1.212	1.442	<0.001
**Primary Insurance Payer**				
** • Medicare**	Ref	Ref	Ref	Ref
** • Medicaid**	1.139	1.014	1.279	0.029
** • Private Insurance**	1.216	1.128	1.310	<0.001
** • Self-Pay**	1.259	1.013	1.565	0.038
** • No Charge**	1.425	0.963	2.109	0.077
** • Others/Missing**	1.840	1.592	2.126	<0.001
**Region**				
** • Northeast**	Ref	Ref	Ref	Ref
** • Midwest**	0.657	0.568	0.760	<0.001
** • South**	0.875	0.774	0.988	0.031
** • West**	0.748	0.643	0.870	<0.001
**Charlson Comorbidity Index^a^**				
** • = <2**	Ref	Ref	Ref	Ref
** • 3**	1.564	1.132	2.160	0.007
** • 4**	1.676	1.230	2.286	0.001
** • > = 5**	2.619	1.950	3.519	<0.001
**Whipple Procedure**				
** • No**	Ref	Ref	Ref	Ref
** • Yes**	0.636	0.545	0.741	<0.001
**Palliative Care Encounter**				
** • No**	Ref	Ref	Ref	Ref
** • Yes**	6.517	5.982	7.101	<0.001

^a^Age-adjusted and weighted.

**Fig 2:**
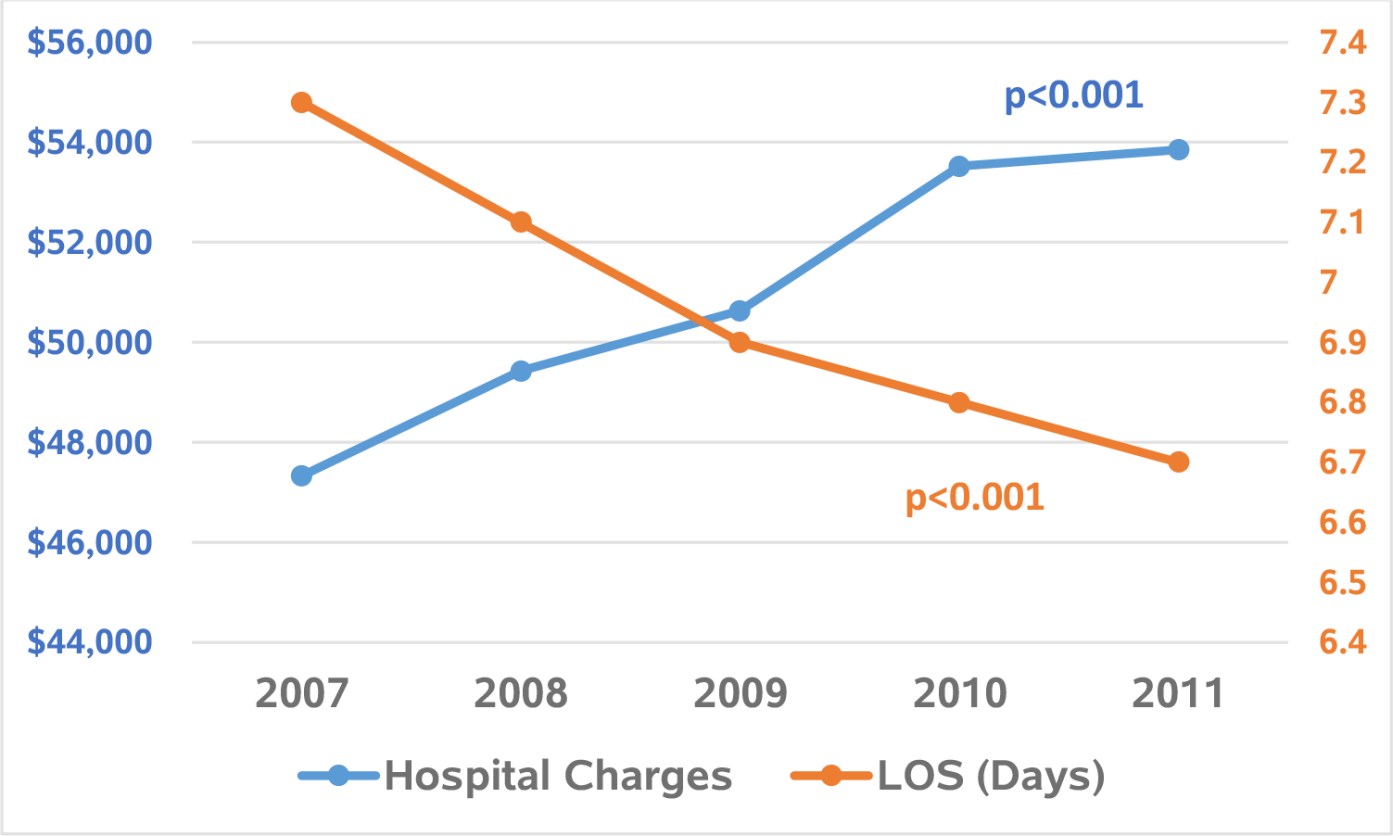
Trends in mean hospital charges and length of stay (LOS) associated with admissions with pancreatic cancer.

Length of stay (LOS) decreased from 7.3 in 2007 to 6.7 in 2011 (p<0.001) (Table 2 and Fig 2). After adjustment to 2011 U.S. dollars using data from the U.S. Bureau of Labor Statistics, the mean hospital charges increased room $47,331 in 2007 to $53, 854 in 2011 (p = 0.01) (Table 2 and Fig 2). The total adjusted hospital charges nationally increased by 34% from 3.8 billion dollars in 2007 to 5.1 billion dollars in 2011 with an average of 4.5 billion dollars per year. We obtained the similar decreasing trend in LOS and increasing trend in hospital charges even with the exclusion of those patients who died in the hospital during index hospitalization (Table 2).

## Discussion

Our study shows improved mortality for hospitalized patients in relation with pancreatic cancer. A decrease in mortality due to pancreatic cancer is immensely important given that incidence nearly parallels mortality rate, and the projected future burden of disease is immense [2]. Similar observations were reported by Wang et al and Kamisawa et al [10, 14]. The possible explanation of our findings are including but not limited to, earlier detection, advances in adjuvant chemotherapy regimens and surgical technique, and further understanding of the molecular deviations contributing to the development of pancreatic cancer [10, 14–16]. Although improved in-hospital mortality from pancreatic cancer was also shown by a previous study [17], it did not elicit the independent predictors of mortality.

The National Cancer Institute declares the estimated incidence for 2016 as 14.1/100,000; a rise from 12.1/100,000 in 2005 [18]. Enlarging projections are likely due to an increase in the number of patients >65 years of age and an increasing number of diagnoses among minorities, who are not only growing in number but may also have a greater incidence of pancreatic cancer [4, 19, 20]. Lastly, there has been limited advancement in screening strategies of pancreatic cancer [4]. Due to the lack of better screening modalities, pancreatic cancers are diagnosed at late stage. Our study showed significant hospital burden from pancreatic cancer. The findings of our study suggest an 18% increase in hospitalization with pancreatic cancer from 2007–2011, indicating the burden of this disease is significant and implying a considerable increase in incidence over the past 5 years. Our findings regarding increased hospitalizations due to pancreatic cancer help validate the statistical projections of the future burden of pancreatic cancer in the United States [4].

Siegel et al also states the 5-year survival between 2005 to 2011 was only 8%, which is a slight improvement in the last 20 years [2]. However, detailed demographics of these mortalities are seldom reported.

Our study successfully showed demographic details like African American and male population at higher risk compared to Caucasian and Hispanics and females. Similar observations were noted in the National Cancer Institute data demographics report [18, 21]. Advanced age has been associated with higher mortality in pancreatic cancer patients [18]. Smith et al noted 73% change in the incidence of pancreatic cancer in patients >65 years of age from 2010 to 2030 [19]. Given the United States demographic shift of increasing population >65 years of age, the projected increasing percentage of patients who will be diagnosed with pancreatic cancer in the future is alarming [4]. Our detailed multivariate analysis also found higher mortality rates amongst individuals with a primary insurance payer other than Medicare. While mortality inequalities exist for all cancers regarding insurance status of the patient, the most important factor in the future to disparage disparities in healthcare outcomes between different insurance coverages will be making quality care accessible to all segments of the population [22].

Although our study showed that the patients who underwent the Whipple procedure (pancreatoduodenectomy) conferred a lower mortality with an odds ratio of 0.64 relative to those who had not undergone the procedure, it will be erroneous to conclude it from the current database since it does not provide any patient identifiers and therefore patients cannot be tracked in a linear fashion to confirm the mortality benefit beyond the scope of the database. It is also worth mentioning that only 15–20% of pancreatic cancer patients are candidates for surgical treatment [5, 6]. Our study found that 6% of admissions had Whipple surgery, suggesting a very limited scope of viable candidates. In a separate analysis, we found that the mean CCI in patients undergoing Whipple surgery was 7.42 **±** 0.11 versus 8.84 **±** 0.02 in patients not undergoing Whipple surgery. It could mean patients who underwent Whipple surgery were those who likely had lower comorbidity burden as evidenced by lower CCI to better withstand surgery.

Perhaps in recent years both improvement in the Whipple procedure in terms of technique and surrounding treatment and management, along with patient selection, played a role in reducing mortality. On sub-group analysis, we found that the number of patients undergoing Whipple surgery increased from 5.5% in 2007 to 6.8% in 2011, with overall increase by 24%. Of particular importance, newer chemotherapy regimens including gemcitabine alongside radiotherapy regimen refinements have been demonstrated to be more effectual than previous treatment regimens [9].

CCI also acts as a very strong predictor of the likelihood of admission based on our study of admission characteristics, which show an overwhelming majority of patient admissions with CCI score ≥5. The increased risk of mortality in patients with a higher CCI score was statistically significant, as the odds ratio on an index score of ≥5 was found to be 2.62. Our analysis helped validate the use of CCI score as an accurate predictor of mortality in patients with pancreatic cancer. Furthermore, Cascinu et al highlighted the significant contributions of adjuvant chemotherapy post-operatively, chemotherapy and chemo-radiotherapy to morbidity, which is worth noting in relation to our predictions of mortality with CCI score [8, 9]. Further research is suggested to document the interplay between assessment of CCI in terms of timing of surgical procedures, adjuvant chemotherapy pre/post-operatively and chemo-radiotherapy. According to our analysis, mortality was also reduced in patients from the Midwest region of the United States (OR 0.7, 95% CI 95% 0.6–0.8). While Wang et al reported geographic variations in initial hospitalization due to pancreatic cancer, their analyses reported similar 1-year mortality rates across the entire United States [10]. While our study included a range of insurance coverages, Wang et al inclusion criteria included only Medicare patients, which may have accounted for the differing mortality rates found between the two studies [10]. From our findings, regional healthcare enhancements can be focused on territories determined to have elevated mortality rates, and further studies may help elucidate regional differences in healthcare contributing to increased or decreased mortality rates. These principles can also be applied to regions with higher 1-year hospitalization rates due to pancreatic cancer.

Our finding that palliative care was significantly associated with mortality is understandably obvious since the patients enrolled in palliative care are possibly the sickest ones and anticipating death given the dismal prognosis of the pancreatic cancer.

LOS among those who survived index admission also decreased between 2007–2011 by 0.6 days per admission (p<0.001), while the mean hospital charge increased by 24%, indicating there have been improvements in patient outcomes alongside more rapid recovery times however at significant financial burdens. Despite the decrease in LOS, the hospital charges increased possibly due to increased use of treatment modalities (chemotherapies, surgeries, etc.) and increased utilization of healthcare resources which translated into significant decrease in mortality. According to Wang et al., LOS decreased from 10.7 days in 2000 to 9.1 days in 2010 [10]. Both ours and Wang’s studies provide statistically significant evidence that LOS due to pancreatic cancer is decreasing. Accordingly, longer LOS in Wang et al. may be due to the fact that the study was limited to Medicare patients, which historically are less healthy than managed care populations [10]. In our study, total hospital charges due to pancreatic cancer was found to be $4.5 billion dollars per year. With projected increases in the number of diagnoses, the economic impact of pancreatic cancer in the future is substantial.

Our study has several limitations inherent to observational epidemiological studies. Our study was limited by its retrospective nature and its use of ICD-9-CM codes for the diagnosis. Many confounders that could influence the outcomes were adjusted in our study. Since our study includes only the hospitalized patients, the results might be little different from that from the outpatient setting, where the patients tend to be less severely ill. Decreasing in-hospital mortality may not exactly reflect actual decreasing mortality from pancreatic cancer since many patients possibly going to hospice or receiving treatment as outpatients could not be captured in the NIS database. In addition, the limitation of the NIS is that isolated admissions as opposed to isolated patients are documented potentially introduces the possibility that patients with recurrent admission may be counted as multiple events in our study.

## Conclusions

In conclusion, pancreatic cancer in the United States represents a very significant burden of disease in the future, both somatically and economically. The additional findings as outlined in our analysis are significant in directing supplementary measures in reducing mortality by tending to the modifiable predictors of mortality–primary insurance payer and regional healthcare improvements, as well as treating and preventing co-morbidities in pancreatic cancer patients. Although unmodifiable predictors of mortality in our analysis, including sex, race and age, were determined to be significant in predicting mortality, preventative measures and screening may be applied in the future to limit morbidity and mortality due to these elements. Given the alarming projected increase in pancreatic cancer diagnoses and pancreatic cancer related deaths, such information may have profound implications regarding future demographics to focus research, screening or preventative measures accordingly. Various groups and bills, including the Pancreatic Cancer Action Network and the Recalcitrant Cancer Research Act of 2013, have produced enhancements in the contemporary approach and rapid advances in research towards improving the survival of those with pancreatic cancer [23, 24]. Conceivably, similar and additional actions will be necessary in the future.
